# Supplementary material for: Metaproteogenomic Profiling of Chemosynthetic Microbial Biofilms Reveals Metabolic Flexibility During Colonization of a Shallow-Water Gas Vent
Source: Front Microbiol. 2021 Apr 6;12:638300. doi: 10.3389/fmicb.2021.638300 (PMC8056087; doi:10.3389/fmicb.2021.638300)
Supplement: Supplementary file 1 [file Data_Sheet_1.docx]

Supplementary Table 1. Metagenomes: Normalized abundances of key genes involved in carbon fixation, nitrogen & sulfur metabolism, oxygen respiration, and heavy metal detoxification pathways expressed as transcripts (reads) per million (TPM).

| Gene | TPM | |
| --- | --- | --- |
|  | Young Filaments (YF) | Established Filaments (EF) |
| ATP-citrate lyase (aclAB) | 681.5 | 210.34 |
| Fumarate reductase (frdAB) | 1331.01 | 115.24 |
| Pyruvate ferredoxin oxidoreductase (porABDG) | 1141.07 | 550.12 |
| Phosphoribulokinase (prk) | 99.47 | 207.22 |
| Ribulose-bisphosphate carboxylase (rbcSL) | 182.77 | 408.48 |
| Nitrate reductase (napAB) | 381.01 | 153.62 |
| Nitrate reductase (narGHI) | 21.55 | 86.98 |
| Nitric oxide reductase (norB) | 1.35 | 3.72 |
| Nitrite reductase (nirBD) | 312.33 | 498.28 |
| Nitrite reductase, cytochrome c-552 (nrfA) | 0.37 | 4.92 |
| Nitrite reductase, NO-forming (nirK) | 0.77 | 7.69 |
| Nitrite reductase, NO-forming (nirS) | 2.23 | 4.81 |
| Nitrogenase (nifDHK) | 499.47 | 325.34 |
| Nitrous-oxide reductase (nosZ) | 6.71 | 12.34 |
| Adenylylsulfate reductase (aprAB) | 17.5 | 26.49 |
| Polysulfide reductase (psrA) | 2.61 | 11.83 |
| Sox system (soxXYZABCD) | 2553.8 | 2308.16 |
| Sulfate adenylyltransferase (sat) | 237.44 | 255.85 |
| Sulfide dehydrogenase (fccAB) | 635.6 | 1402.89 |
| Sulfide:quinone oxidoreductase (sqr) | 375.84 | 843.84 |
| Sulfide dehydrogenase | 52.65 | 2.22 |
| Sulfite reductase (dsrAB) | 76.72 | 210.2 |
| Sulfur oxygenase/reductase (sor) | 0 | 38.43 |
| Putative Sulfur reductase (nsr) | 323.84 | 394.22 |
| Cytochrome c oxidase, aa3-type (coxABCD) | 834.02 | 665.43 |
| Cytochrome c oxidase, cbb3-type (ccoNOPQ) | 944.63 | 1026.12 |
| Cytochrome bd ubiquinol oxidase (cydAB) | 284.87 | 392.7 |
| Arsenate reductase (arsC) | 527.49 | 543.82 |
| Mercuric reductase (merA) | 502.49 | 174.16 |
| Selenate detoxification (dedA) | 0.69 | 15.14 |

Supplementary Table 2. Metaproteomes: Normalized abundances of key proteins involved in carbon fixation, nitrogen & sulfur metabolism, oxygen respiration, and heavy metal detoxification pathways expressed as a percentage of total proteins.

| Protein | Percent | |
| --- | --- | --- |
|  | Young Filaments (YF) | Established Filaments (EF) |
| ATP-citrate lyase (AclAB) | 1.202 | 0.545 |
| Fumarate reductase (FrdAB) | 0.008 | 0.003 |
| Pyruvate ferredoxin oxidoreductase (PorABDG) | 3.825 | 1.677 |
| Phosphoribulokinase (PRK) | 0.081 | 0.267 |
| Ribulose-bisphosphate carboxylase (RbcSL) | 0.623 | 0.846 |
| Nitrate reductase (NarGHI) | 0.014 | 0.025 |
| Nitrite reductase (NirBD) | 0.103 | 0.329 |
| Nitrogenase (NifDHK) | 0.002 | 0.354 |
| Adenylylsulfate reductase (AprAB) | 0.006 | 0.015 |
| Sox system (SoxXYZABCD) | 1.85 | 1.635 |
| Sulfate adenylyltransferase (SAT) | 0.085 | 0.18 |
| Sulfide dehydrogenase (FccAB) | 0.351 | 0.72 |
| Sulfide:quinone oxidoreductase (Sqr) | 0.336 | 0.42 |
| Sulfite reductase (DsrAB) | 0.399 | 0.639 |
| Sulfur oxygenase/reductase (Sor) | 0.013 | 0.06 |
| Putative Sulfur reductase (Nsr) | 1.799 | 0.301 |
| Cytochrome c oxidase, aa3-type (CoxABCD) | 0.397 | 0.083 |
| Cytochrome c oxidase, cbb3-type (CcoNOPQ) | 0.357 | 0.469 |
| Cytochrome bd ubiquinol oxidase (CydAB) | 0.014 | 0.073 |
| Arsenate reductase (ArsC) | 0 | 0.003 |
| Selenate detoxification (DedA) | 0.008 | 0.004 |
